# Spontaneous isolated superior mesenteric artery dissection

**DOI:** 10.11604/pamj.2020.37.147.22922

**Published:** 2020-10-13

**Authors:** Hirohisa Fujikawa, Taigo Sato

**Affiliations:** 1 Department of Medical Education Studies, International Research Center for Medical Education, Graduate School of Medicine, The University of Tokyo, 7-3-1 Hongo, Bunkyo-ku, Tokyo 113-0033, Japan,; 2 Department of Internal Medicine, Suwa Central Hospital, 4300 Tamagawa, Chino, Nagano 391-8503, Japan

**Keywords:** Superior, mesenteric artery, dissection, abdominal pain, computed tomography angiography

## Image in medicine

A 64-year-old woman presented with a 2-day history of epigastralgia. Abdominal exam was soft with severe epigastric discomfort to deep palpation. A non-contrast-enhanced computed tomography (CT) demonstrated a dilated superior mesenteric artery (SMA) (A). What is your diagnosis? A contrast-enhanced CT revealed dissection of the SMA (B, C). We diagnosed spontaneous isolated SMA dissection (SISMAD). Because no findings indicating mesenteric ischemia or concomitant dissection of the aorta were present, she was managed conservatively with antiplatelet and antihypertensive treatment. Her abdominal pain resolved, and she was discharged home two weeks after admission. SISMAD is defined as SMA dissection without aortic dissection. SISMAD is rare but increasingly becoming recognized due to the development of imaging technology. It may occur secondary to atherosclerosis, cystic medial necrosis, elastic tissue diseases, fibromuscular dysplasia or trauma, but many cases are idiopathic. CT angiography is a good diagnostic tool and can detect any bowel infarction or vessel-related complications. Treatment options include conservative, endovascular or surgical treatment. Endovascular therapy is suggested for patients with persistent or recurrent symptoms, whereas surgery should be performed immediately for patients with intestinal necrosis, arterial rupture or failed endovascular management. The clinical spectrum of SISMAD ranges from asymptomatic incidental findings to catastrophic bowel ischemia or SMA aneurysm rupture, thus patients can visit diverse departments. SISMAD may be unrecognized if a clinician does not suspect or order appropriate imaging modalities. Rapid recognition and treatment can be crucial for patient survival, so clinicians should consider SISMAD as a possible differential diagnosis of abdominal pain.

**Figure 1:**
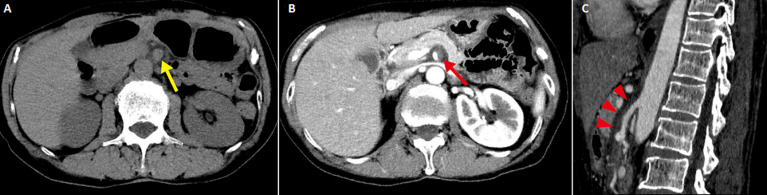
A) non-contrast-enhanced computed tomography image of the abdomen revealing the dilation of the superior mesenteric artery (yellow arrow); B, C) contrast-enhanced computed tomography image of the abdomen depicting the dissection of the superior mesenteric artery (red arrow and arrowheads)

